# Key features of puberty onset and progression can help distinguish self-limited delayed puberty from congenital hypogonadotrophic hypogonadism

**DOI:** 10.3389/fendo.2023.1226839

**Published:** 2023-08-28

**Authors:** Yuri Aung, Vasilis Kokotsis, Kyla Ng Yin, Kausik Banerjee, Gary Butler, Mehul T. Dattani, Paul Dimitri, Leo Dunkel, Claire Hughes, Michael McGuigan, Márta Korbonits, George Paltoglou, Sophia Sakka, Pratik Shah, Helen L. Storr, Ruben H. Willemsen, Sasha R. Howard

**Affiliations:** ^1^ Centre for Endocrinology, William Harvey Research Institute, Queen Mary, University of London (QMUL), London, United Kingdom; ^2^ Department of Paediatric Endocrinology, Royal London Children’s Hospital, Barts Health NHS Trust, London, United Kingdom; ^3^ Department of Paediatrics, Barking, Havering and Redbridge University Hospitals NHS Trust, London, United Kingdom; ^4^ Department of Paediatric and Adolescent Endocrinology, University College London Hospital NHS Foundation Trust, London, United Kingdom; ^5^ UCL Great Ormond Street (GOS) Institute of Child Health, University College London, London, United Kingdom; ^6^ Department of Paediatric Endocrinology, Great Ormond Street Hospital for Children NHS Foundation Trust, London, United Kingdom; ^7^ Department of Paediatric Endocrinology, Sheffield Children’s Hospital NHS Foundation Trust, Sheffield, United Kingdom; ^8^ Department of Paediatrics, Countess of Chester NHS Foundation Trust, Chester, United Kingdom; ^9^ Department of Endocrinology, Barts Health NHS Trust, London, United Kingdom; ^10^2nd Department of Paediatrics, National and Kapodistrian University of Athens (NKUA), “P. & A. Kyriakou” Children’s Hospital, Athens, Greece; ^11^ Department of Paediatric Endocrinology, Evelina Children’s Hospital, Guys and St Thomas’ NHS Foundation Trust, London, United Kingdom

**Keywords:** puberty, idiopathic hypogonadotrophic hypogonadism, delayed puberty, self-limited delayed puberty, Kallmann syndrome, hypogonadism

## Abstract

**Introduction:**

Delayed puberty (DP) is a frequent concern for adolescents. The most common underlying aetiology is self-limited DP (SLDP). However, this can be difficult to differentiate from the more severe condition congenital hypogonadotrophic hypogonadism (HH), especially on first presentation of an adolescent patient with DP. This study sought to elucidate phenotypic differences between the two diagnoses, in order to optimise patient management and pubertal development.

**Methods:**

This was a study of a UK DP cohort managed 2015-2023, identified through the NIHR clinical research network. Patients were followed longitudinally until adulthood, with a definite diagnosis made: SLDP if they had spontaneously completed puberty by age 18 years; HH if they had not commenced (complete, cHH), or had commenced but not completed puberty (partial, pHH), by this stage. Phenotypic data pertaining to auxology, Tanner staging, biochemistry, bone age and hormonal treatment at presentation and during puberty were retrospectively analysed.

**Results:**

78 patients were included. 52 (66.7%) patients had SLDP and 26 (33.3%) patients had HH, comprising 17 (65.4%) pHH and 9 (34.6%) cHH patients. Probands were predominantly male (90.4%). Male SLDP patients presented with significantly lower height and weight standard deviation scores than HH patients (height p=0.004, weight p=0.021). 15.4% of SLDP compared to 38.5% of HH patients had classical associated features of HH (micropenis, cryptorchidism, anosmia, etc. p=0.023). 73.1% of patients with SLDP and 43.3% with HH had a family history of DP (p=0.007). Mean first recorded luteinizing hormone (LH) and inhibin B were lower in male patients with HH, particularly in cHH patients, but not discriminatory. There were no significant differences identified in blood concentrations of FSH, testosterone or AMH at presentation, or in bone age delay.

**Discussion:**

Key clinical markers of auxology, associated signs including micropenis, and serum inhibin B may help distinguish between SLDP and HH in patients presenting with pubertal delay, and can be incorporated into clinical assessment to improve diagnostic accuracy for adolescents. However, the distinction between HH, particularly partial HH, and SLDP remains problematic. Further research into an integrated framework or scoring system would be useful in aiding clinician decision-making and optimization of treatment.

## Introduction

Delayed puberty (DP) is defined as the onset of puberty 2 to 2.5 standard deviations (SD) later than the general population, and is typically classified as the absence of breast development by age 13 years in girls, or testicular enlargement ≥4ml by age 14 years in boys (1). It affects 2% of adolescents and is more commonly a presentation in adolescence in boys (2). This predominance is thought to be due to a combination of referral bias in boys with short stature due to DP (3, 4), with a later onset of the pubertal growth spurt in boys (5), as well as earlier detection of the first signs of puberty in girls.

The commonest aetiology of DP is self-limited delayed puberty (SLDP, also known as constitutional delay of growth and puberty), with a temporary picture of isolated hypogonadotrophic hypogonadism which resolves with time or a short course of sex steroid therapy (1, 6). SLDP is found to be the underlying cause in 70% of males and 32% of females presenting with DP (7). Estimates of the heritability of SLDP range from 50-80% (7, 8), with a previous familial study suggesting that up to 80% of male and 75% of female SLDP patients have affected first-degree relatives (5). Typically, SLDP is inherited in an autosomal dominant pattern with or without complete penetrance (9). Although SLDP was previously considered a benign variant in pubertal timing, it has now been linked to adverse consequences in later life in terms of health, psychological and socioeconomic impact (10–12). To this end, treatment with sex steroids may be appropriate to improve later outcomes (7). However, the role of hormonal treatment in SLDP is not standardised despite national guidelines, for example from the British Society for Paediatric Endocrinology and Diabetes, with varying physician preferences (7, 10).

Other aetiologies of DP include hypergonadotropic hypogonadism secondary to primary gonadal disorders, functional hypogonadism due to chronic illness or nutritional deficit and idiopathic hypogonadotrophic hypogonadism (HH). Whereas functional and hypergonadotropic hypogonadism may be easier to formally diagnose (1, 13), the differentiation between HH and SLDP is more complex, and is often a challenge for paediatric endocrinologists. Defined as a state of gonadotrophic deficiency which disrupts the hypothalamic-pituitary-gonadal axis, HH can then be further classified into complete and partial subtypes, where either puberty was never initiated (complete, cHH) or started but subsequently arrested (partial, pHH) (14). Whilst the original description of this condition was of gonadotropin deficiency secondary to underproduction of gonadotropin-releasing hormone (GnRH) from the hypothalamus, the aetiology also includes a mutation in the GnRH receptor gene leading to GnRH resistance.

The severity of DP observed varies according to the degree of gonadotrophic deficiency (14), with an extensive clinical spectrum ranging from extreme forms with classical features, to milder forms that are difficult to distinguish from and often overlap with SLDP (1). Up to 20% of patients with HH may also have periods of reversal of their phenotype, a little understood phenomenon where patients become eugonadal for a variable length of time (15).

Regardless, all patients with HH may benefit from initiation of hormonal treatment as early as possible. Thus, whilst SLDP has often been considered a diagnosis of exclusion, typically made retrospectively once puberty is completed, it is vital to make a diagnosis of HH as promptly as possible to prevent delay in optimising treatment (16). Even for patients with a final diagnosis of SLDP, delay in diagnosis and lack of clarity about future pubertal progression contributes to the psychological distress and social difficulties often experienced by this patient group (10, 12). Treatment for patients with SLDP may be indicated to facilitate secondary sexual characteristics, increase height and to promote endogenous hypothalamic-pituitary-gonadal (HPG) axis function, but also to address the psychological distress that can be felt from these patients being delayed compared to their peers.

Previous research into distinguishing between SLDP and HH have identified several potential differentiating markers (17). From a phenotypic perspective, there are key syndromic and physical features more often seen in permanent hypogonadotrophic states (e.g. micropenis, cryptorchidism (particularly bilateral), anosmia, cleft lip/palate, renal and skeletal anomalies) (17). Growth patterns within the first 5 years of life may vary between the two conditions, with SLDP patients tending to show height and weight deficit earlier on in childhood than in patients with HH (16). HH has also been associated with several biochemical markers, including lower serum concentrations of inhibin B and anti-mullerian hormone (AMH) (6, 17, 18) and a reduced response to hCG and/or GnRH stimulation testing (17, 19, 20). From a genotypic perspective, multiple genes have been implicated in the pathogenesis of these two conditions, with evidence suggesting that targeted exome sequencing may be of use in aiding differential diagnosis between SLDP and HH (13). However, the literature on phenotypic variation between the two aetiologies remains sparse, with variable replication, and often limited to single-centre populations, with no overall conclusive method of differentiation (17). Therefore, this study utilised longitudinal data to analyse the phenotypic variance in our multi-centre cohort of SLDP and HH patients who had undergone long-term follow up until early adulthood, with the aim of identifying new markers of differentiation or clarifying associations previously presented in the literature.

## Methods

This was a retrospective study of paediatric and young adult patients with central delayed puberty who had completed puberty, identified through the NIHR clinical research network portfolio study (CPMS ID 39730) collating patients evaluated for delayed puberty within the United Kingdom from 2015 to 2023. Six paediatric endocrine centres contributed patients to the study: Royal London Children’s Hospital Barts Health NHS Trust, Great Ormond Street Hospital for Children NHS Foundation Trust, University College London Hospitals NHS Foundation Trust, Countess of Chester Hospital NHS Foundation Trust, Guy’s and St Thomas’ NHS Foundation Trust and Sheffield Teaching Hospitals NHS Foundation Trust. Ethical approval was granted by the London–Chelsea NRES committee and the UK NHS Health Research Authority (13/LO/0257), with all participants or parents of child patients providing written informed consent.

Delayed puberty was defined as attaining Tanner stage G2 or B2 at 2 standard deviations later than the population average, thus a threshold of later than 14 years for boys and 13 years for girls. Arrested or stalled puberty, with spontaneous entry into puberty with attainment of G2 or B2, followed by slow or absent further progression, was assessed via Copenhagen Puberty Study nomograms (in males testes ≤6 mL at 15.0 years, and/or ≤8 mL at 16.3 years or older) (10). Patients were all followed longitudinally until at least 18 years of age and a definite diagnosis of SLDP or HH had been made. Individuals were diagnosed with SLDP if they had attained Tanner stage G4 or B4 by this age, either spontaneously or with a short course of sex steroids. If Tanner G4 or B4 had not been reached by 18 years, they were either diagnosed with complete HH (cHH) if they had not commenced spontaneous puberty at all, or partial HH (pHH) if they had entered puberty but then arrested. Patients with delayed puberty due to functional hypogonadism, including due to chronic inflammatory diseases, under-nutrition or excessive exercise (diagnosed by detailed medical history of renal, cardiac, respiratory, rheumatological or haematological disorders, physical examination with multi-system review alongside body mass index, and routine laboratory investigations for inflammatory markers, full blood count, renal, liver and bone profile) or hypergonadotropic hypogonadism were excluded from this study, as were patients with central hypogonadism secondary to cancer or cancer treatment, and patients with complex syndromes.

Phenotypic data were manually extracted from clinical information and investigations held electronically and in patient notes, including data pertaining to auxology, Tanner staging, testicular volume, blood biochemistry (luteinising hormone, LH; follicle-stimulating hormone, FSH; oestradiol; testosterone; inhibin B; AMH), bone age, and hormonal treatment. All data were reviewed and analysed independently by two researchers. The application Auxology (KIGS, Pfizer) was used to complete auxology for all anthropometric characteristics including mid-parental height (MPH) and height velocity (HV). Adult height was calculated as when height velocity fell to <1cm/year. LH and FSH were measured via ICMA. Where blood test results were below the limit of detection (LOD), 50% of the LOD was used for subsequent statistical analysis (21). Bone age was determined via BoneXpert using TW3 estimates.

A confirmed family history of SLDP was defined as maternal age of menarche above the age of 15 years, or an immediate family member having either hormonal treatment for delayed puberty or delayed pubertal onset beyond 13 years in females and 14 years in males. A possible family history was defined as any other mention of suspected delayed puberty (e.g. late growth spurt, late voice break or other pubertal phenotypes) in a family member.

Included ‘red flag’ signs for HH were micropenis or cryptorchidism (unilateral or bilateral), anosmia or hyposmia, cleft lip or palate, synkinesis, renal anomalies, and limb anomalies. Data at presentation were extracted from patients’ first referrals or first consultations with tertiary endocrinology services where available. Pubertal onset data were classified as when testicular volume reached 4ml in males (G2), or Tanner stage B2 was documented in females. End of puberty data were extracted at the point where stage B4 or G4 was documented.

Statistical analysis was completed using IBM SPSS version 28 and R, with mean values displayed ± standard deviation and a P value of <0.05 considered statistically significant. Z-scores (SDS) for weight and height parameters were used to standardise weight and height data for age. Graphs were created using GraphPad Prism 8.

## Results

### Demographic characteristics of the delayed puberty cohort are similar in patients with SLDP and HH

The cohort included 78 patients with delayed puberty, of whom 52 patients (66.7%) had a final diagnosis of SLDP and 26 (33.3%) were diagnosed with HH. Of the patients with HH, 17 (65.4%) had a diagnosis of partial HH (pHH) and 9 (34.6%) had complete HH (cHH). Both SLDP and HH populations were predominantly male (90.4% and 84.6% respectively), of heterogenous ethnicity with the largest minority of white Caucasian heritage (46.2% SLDP, 50.0% HH). There were no significant differences in gender (p=0.45) or ethnicity (p=0.96) between the two diagnostic groups. SLDP patients were followed up for an average of 5.19 ± 2.71 years from presentation and HH patients for 5.14 ± 3.32 years (p=0.99). A summary of phenotypic characteristics can be found in **Table 1**.

**Table 1:** Phenotypic data at presentation of SLDP and HH patients from the DP cohort.

	SLDP (n=52)		HH (n=26)		P value* (SLDP vs HH)	pHH (n=17)		cHH (n=9)		P value** (pHH vs cHH)
Gender (n, %)										
Male	47	90.4	22	84.6	0.452	14	82.4	8	88.9	
Female	5	9.6	4	15.4		3	17.6	1	11.1	
Ethnicity (n, %)										
Caucasian	24	46.2	13	50.0	0.963	6	35.3	7	77.8	
Asian	6	11.5	4	15.4		3	17.6	1	11.1	
Black	5	9.6	3	11.6		3	17.6	0		
Other	4	7.7	5	19.2		4	23.5	1	11.1	
Unknown	13	25.0	1	3.8		1	5.9	0		
**FU duration** (years ± SD)	5.19	2.71	5.14	3.32	0.989	4.61	3.23	6.25	3.43	0.517
FH of DP (n, %)										
Yes	12	23.1	7	26.9		6	35.3	1	11.1	
Possible	26	50.0	4	15.4		4	23.5	0		
No	14	26.9	15	56.7	**0.007**	7	41.2	8	88.9	**0.003**
**Consanguinity** (n, %)	3	5.8	2	7.7		0		2	22.2	
**Maternal age at menarche** (n=28, mean ± SD)	14.3	1.80	13.2	2.21	0.217	13.6	2.12	11.8	2.36	0.187
MPH (cm ± SD)										
Male (n=37)	174.7	7.92	175.7	7.06	0.635	175.3	8.22	176.6	5.16	0.889
Female (n=5)	162.4	4.96	161.3	7.35		156.1	0	166.5	0	

FU, follow-up; FH, family history; MPH, mid-parental height.

*comparison of SLDP vs HH by student t-test. **comparison of SLDP vs pHH vs cHH by one-way Anova.

Absent p values are due to insufficient numbers for comparison. P values <0.05 are highlighted in bold font.

A family history of delayed puberty was more common in patients with a final diagnosis of SLDP, with 73.1% of SLDP patients having a definite or possible family history of DP, as compared to 43.3% of patients with HH (p=0.001). Family history of delayed puberty was particularly rare in patients with cHH, reported in only 11.1% of this subgroup (p=0.003). The maternal age of menarche was on average one year later in the SLDP group than the HH group, with mean ages of 14.3 ± 1.8 and 13.2 ± 2.2 years respectively, but this difference did not reach significance (p=0.22). Only a small number of patients in each group had maternal age of menarche at age 15 years or above (SLDP, 8 patients; HH, 4 patients). In contrast to a family history of DP, a family history of consanguinity was rare in this cohort in both SLDP patients (5.8%) and in patients with HH (7.7%).

Parental height was not different between the two diagnostic groups, with mean MPH of 174.7 ± 7.9cm in males with SLDP, and 175.7 ± 7.1cm in male patients with HH (p=0.635). Mean MPH was 162.4 ± 5.0cm in female SLDP patients, and 161.3 ± 7.4cm in female HH patients.

### Patients with SLDP are younger, shorter and lighter than patients with HH at presentation

**Figure 1:**
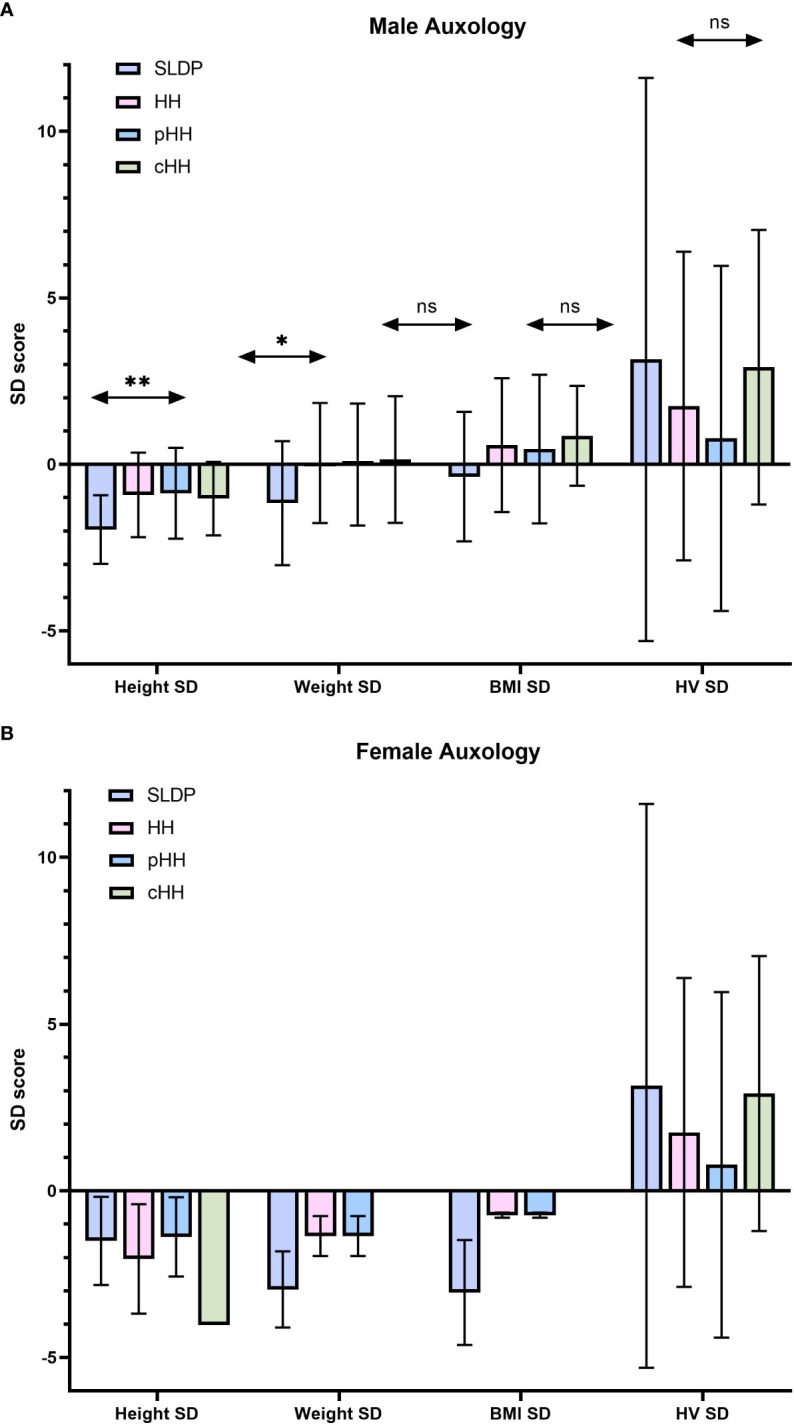
Summary of auxology at presentation in male **(A)** and female **(B)** individuals. Results are presented as mean ± SD. HH group contains both the pHH and cHH groups. SD, standard deviation score; HH, hypogonadotrophic hypogonadism; SLDP, self-limited delayed puberty; pHH, partial hypogonadotrophic hypogonadism; cHH, complete hypogonadotrophic hypogonadism; HV, height velocity. *p = 0.021, **p=0.004. ns, non-significant.

The mean age at presentation in males with SLDP (14.9 ± 1.47 years) was younger that in those with HH (15.7 ± 2.51 years), but this did not reach significance (p=0.09, **Table 2**). However, age at presentation ranged widely from 9.97 to 19.54 years for males across the cohort. Male patients were generally of below average height and weight for age, including for their mid-parental target height (**Figure 1A**). Patients with SLDP were significantly shorter (height SD score for age of -1.96 ± 1.03 as compared to -0.92 ± 1.27 in HH patients; p=0.004) than their counterparts. Patients in the SLDP group were also lighter than those in the HH group (weight SD score for age of -1.17 ± 1.86 as opposed to 0.04 ± 1.80 in HH patients; p=0.02), but BMI SD score for age was not signfiicanctly different between the two groups (p=0.06), suggesting the difference in weight was secondary to the difference in height.

**Table 2:** Auxological and pubertal staging data at presentation of SLDP and HH patients from the DP cohort.

	SLDP (n=44)		HH (n=25)		P value* (SLDP vs HH)	pHH (n=17)		cHH (n=9)		P value** (SLDP vs pHH vs cHH)
Gender (n, %)										
Male	40	90.9	21	84.0	0.389	14	82.4	7	87.5	
Female	4	9.1	4	16.0		3	17.6	1	12.5	
Age (yrs ± SD)										
Male (n=61)	14.9	1.47	15.7	2.51	0.092	16.0	1.90	14.9	3.48	0.185
Female (n=8)	14.6	2.16	14.0	1.69		15.3	2.61	14.5	2.36	
Auxology, Male (SD score ± SD)										
Height SDS (n=56)	-1.96	1.03	-0.92	1.27	**0.004**	-0.87	1.37	-1.03	1.11	**0.014**
Weight SDS (n=56)	-1.17	1.86	0.04	1.80	**0.021**	-0.01	1.83	0.14	1.90	0.071
BMI SDS(n=56)	-0.37	1.95	0.58	2.01	0.058	0.46	2.23	0.86	1.50	0.159
HP SDS (n=31)	-2.09	1.30	-1.70	1.10	0.643	-1.70	1.09	-1.62	1.41	0.882
HV SDS (n=35)	3.15	8.45	1.75	4.63	0.903	0.78	5.18	2.92	4.13	0.731
Auxology, Female (SD score ± SD)										
Height SDS (n=8)	-1.77	1.41	-1.50	1.32		-2.04	1.64	-1.38	1.19	
Weight SDS (n=7)	-2.96	1.14	-1.36	0.60		-1.36	0.60	0		
BMI SDS (n=7)	-3.05	1.57	-0.73	0.08		-0.73	0.08	0		
HV SDS (n=4)	-0.60	0.57	0.71	0.90		0.71	0.90	0		
BA delay Males (years, mean ± SD)										
CA (n=46)	14.9	1.54	15.7	1.80		15.7	1.48	15.7	2.44	
BA (n=47)	12.5	1.07	12.8	1.57		13.5	0.74	13.7	0.52	
CA minus BA (n=47)	2.85	0.95	2.08	1.26	0.432	2.17	1.15	1.96	2.20	0.731
BA delay Female (years, mean ± SD)										
CA (n=6)	11.7	4.45	14.4	2.28		14.4	2.28			
BA (n=6)	9.47	2.29	11.6	1.65		11.6	1.65			
CA minus BA (n=6)	2.53	2.40	2.79	0.70		2.79	0.70			
Testicular volume, Male in ml (n, %)										
<4	5	16.1	10	58.8		5	45.5	5	83.3	
4 to 9	23	74.2	5	29.4		3	36.4	1	16.7	
10 and above	3	9.7	2	11.8		2	18.2	0		
Mean value	5.98	2.63	4.24	2.79	**0.015**	4.91	3.18	3.00	1.38	**0.019**
Genital staging, Male (n, %)										
I	10	27.8	10	52.6	0.085	5	38.5	5	83.3	**0.045**
II	17	47.2	6	31.6		5	38.5	1	16.7	
III	6	16.7	3	15.8		3	23.1	0		
IV	3	8.3	0			0		0		
Pubic hair staging, Male (n, %)										
I	13	50.0	4	26.7	0.265	4	36.4	0	0	0.291
II	11	42.3	10	66.7		6	54.5	4	100.0	
III	2	7.7	0	0		0	0	0	0	
IV	0		1	6.7		1	9.1	0	0	
Axillary hair staging, Male (n, %)										
I	18	81.8	5	38.5	**0.029**	5	55.6	0		**0.007**
II	4	18.2	7	53.8		3	33.3	4	100.0	
III	0		1	7.7		1	11.1	0		
Breast staging, Female (n, %)										
I	2	50.0	2	50.0		2	66.7	0		
II	1	25.0	1	25.0		1	33.3	0		
III	1	25.0	1	25.0		0		1	100.0	
Pubic hair staging, Female (n, %)										
I	0		1	33.3		1	33.3			
II	1	33.3	1	33.3		1	33.3			
III	1	33.3	1	33.3		1	33.3			
IV	1	33.3	0			0				
Axillary hair staging, Female (n, %)										
I	1	33.3	1	25.0		1	33.3	0		
II	2	66.7	2	50.0		1	33.3	1	100.0	
III	0		1	25.0		1	33.3	0		

Staging refers to Tanner stage, SDS, standard deviation score; CA, chronological age; BA, bone age.

*comparison of SLDP vs HH by student t-test. **comparison of SLDP vs pHH vs cHH by one-way Anova.

Absent p values are due to insufficient numbers for comparison. P values <0.05 are highlighted in bold font.

Bone age at presentation was delayed in both male diagnostic groups; whilst there was a greater degree of bone age delay in the SLDP group this did not reach significance (chronological age - bone age in male SLDP patients 2.85 ± 0.95 years; male HH patients 2.08 ± 1.26 years, p=0.43). However, the difference in height SD for chronological age at presentation was not seen when height SD score for bone age was compared between the two groups (height SD score for bone age of 0.02 ± 1.16 in SLDP versus 0.56 ± 1.67 in HH), suggesting that much of the difference seen is due to delay in physical maturation of the SLDP group. There was no significant difference in males at presentation in height velocity SD score for chronological age (p=0.90).

Female mean age at presentation was 14.0 ± 1.69 years for SLDP patients and 15.3 ± 2.61 years for HH patients. Female patients both with SLDP and HH were below average height, weight and BMI at presentation (**Figure 1B**, **Table 2**). Bone age was also delayed in both female populations at presentation, to a similar degree, with a mean delay of 2.53 ± 2.40 years in SLDP patients and 2.79 ± 0.70 years in HH patients.

### Pubertal staging at presentation and associated features of GnRH deficiency differed between patients with SLDP and HH

At presentation, 16.1% of male SLDP patients and 58.8% of male HH patients were pre-pubescent with testicular volume <4ml (**Table 2**). Mean testicular volume at presentation was 5.98 ± 2.63ml in SLDP patients and significantly lower at 4.24 ± 2.79ml in patients with HH (p=0.02); and, as expected for diagnosis, markedly lower in patients with cHH at 3.00 ± 1.38ml, than in patients with pHH at 4.91 ± 3.18ml (p=0.02). Those with pHH as a final diagnosis did not, however, differ significantly in terms of testicular volume at presentation from those with SLDP (p=0.21). Reflecting this, cHH patients were at a lower mean Tanner genital stage at presentation (83.3% G1) than those with pHH (38.5% G1) or SLDP (27.8% G1, p=0.05). In comparison, male cHH patients were at a higher Tanner axillary stage when first seen (100% at stage A2) than pHH patients (33.3% at A2) or SLDP patients (18.2% at A2, p=0.01), a finding that is likely to represent the onset of adrenarche in these patients. There were no significant differences in male Tanner pubic hair staging (p=0.27).

Half of female patients in both SLDP and HH patient groups were pre-pubertal at presentation with Tanner breast stage 1, and pubic and axillary hair staging were similar between the two diagnostic groups (**Table 2**).

As anticipated, classical ‘red flags’ for clinical detection of HH were more common in the patients with a diagnosis of HH rather than SLDP (p=0.02, **Table 3**). Micropenis appeared to be the best discriminator, as it was found in 33.3% of cHH patients, whilst only in 3.8% of SLDP patients and 11.8% of pHH patients (p=0.02). A history of cryptorchidism (all bilateral) was seen in 22.2% of cHH patients, but in 5.8% and 11.5% of SLDP and pHH patients respectively (p=0.18). Anosmia or hyposmia was seen more often, not only in those with cHH (22.2%), but also in those with pHH (17.6%), as compared to SLDP patients (3.8%) (p=0.05). Renal anomalies and synkinesis were rare findings, each found only in one patient with cHH. One patient with SLDP (but no patients with HH) had digit or limb anomalies. One female patient with pHH had a history of cleft palate repair. Overall, 44 SLDP patients (84.6%) and 16 HH patients (61.5%) had no ‘red flag’ signs of HH.

**Table 3:** Associated ‘red flag’ features of HH in the SLDP and HH patients from the DP cohort.

	SLDP (n=52)		HH (n=26)		P value* (SLDP vs HH)	pHH (n=17)		cHH (n=9)		P value** (SLDP vs pHH vs cHH)
HH features (n, %)										
Micropenis	2	3.8	5	19.2	**0.023**	2	11.8	3	33.3	
Cryptorchidism	3	5.8	3	11.5	0.183	1	5.9	2	22.2	
Anosmia	2	3.8	5	19.2	**0.025**	3	17.6	2	22.2	
Cleft lip or palate	0		1			1		0		
Renal anomaly	0		1	3.8		0		1	11.1	
Synkinesis	0		1	3.8		0		1	11.1	
Limb anomaly	1	1.9	0			0		0		
None	44	84.6	16	61.5		11	64.7	5	55.6	
Any	8	15.4	10	38.5	**0.023**	6	35.3	4	44.4	0.065

*comparison of SLDP vs HH by student t-test. **comparison of SLDP vs pHH vs cHH by one-way Anova.

Absent p values are due to insufficient numbers for comparison. P values <0.05 are highlighted in bold font.

### Biochemical markers of LH and inhibin B may aid differentiation, particularly between male patients with SLDP and cHH

**Figure 2:**
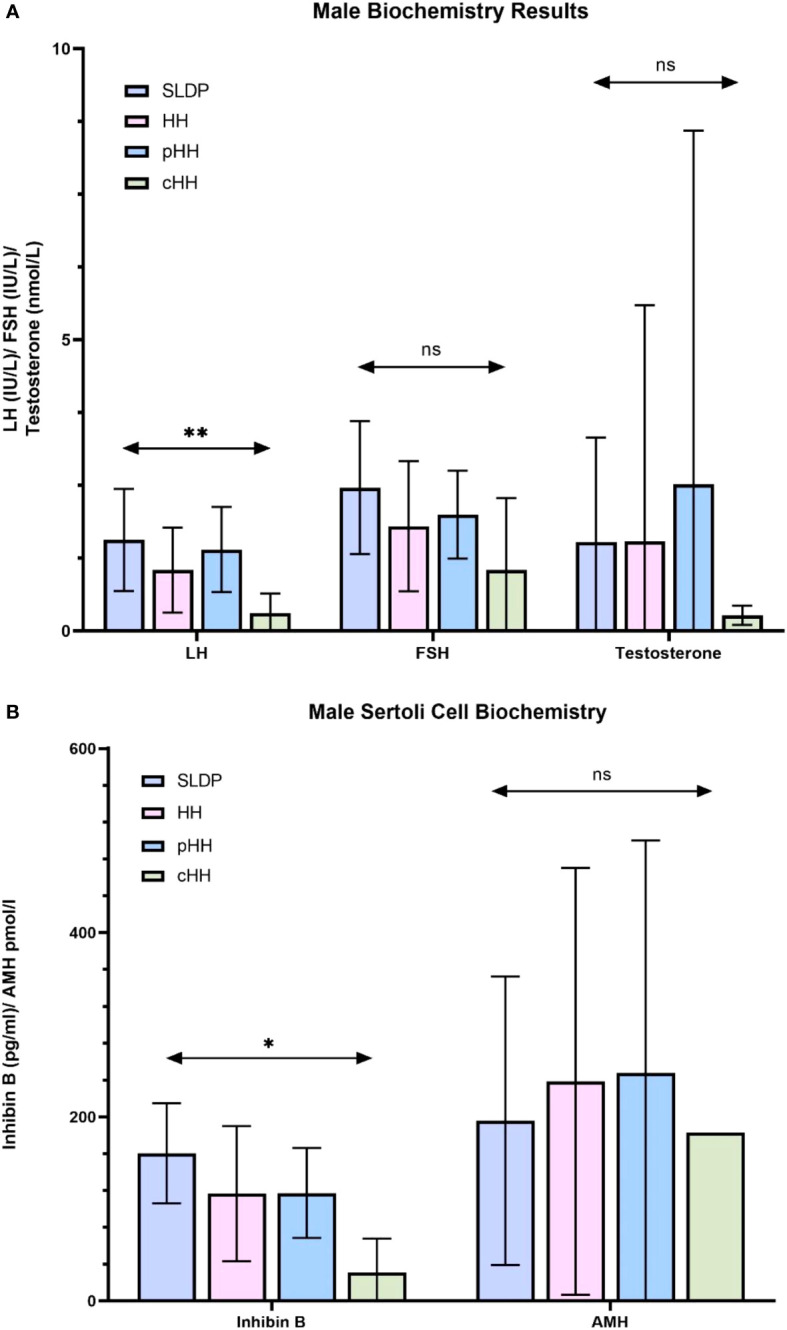
Summary of biochemistry at presentation in male SLDP and HH populations. Results are presented as mean ± SD. HH group contains both the pHH and cHH groups. **(A)** LH, luteinising hormone; FSH, follicle-stimulating hormone, and testosterone. Panel B: inhibin, inhibin **(B)** AMH, anti-mullerian hormone; HH, hypogonadotrophic hypogonadism; SLDP, self-limited delayed puberty; pHH, partial hypogonadotrophic hypogonadism; cHH, complete hypogonadotrophic hypogonadism. *p = 0.014, **p = 0.005. ns, non-significant.

**Figure 3:**
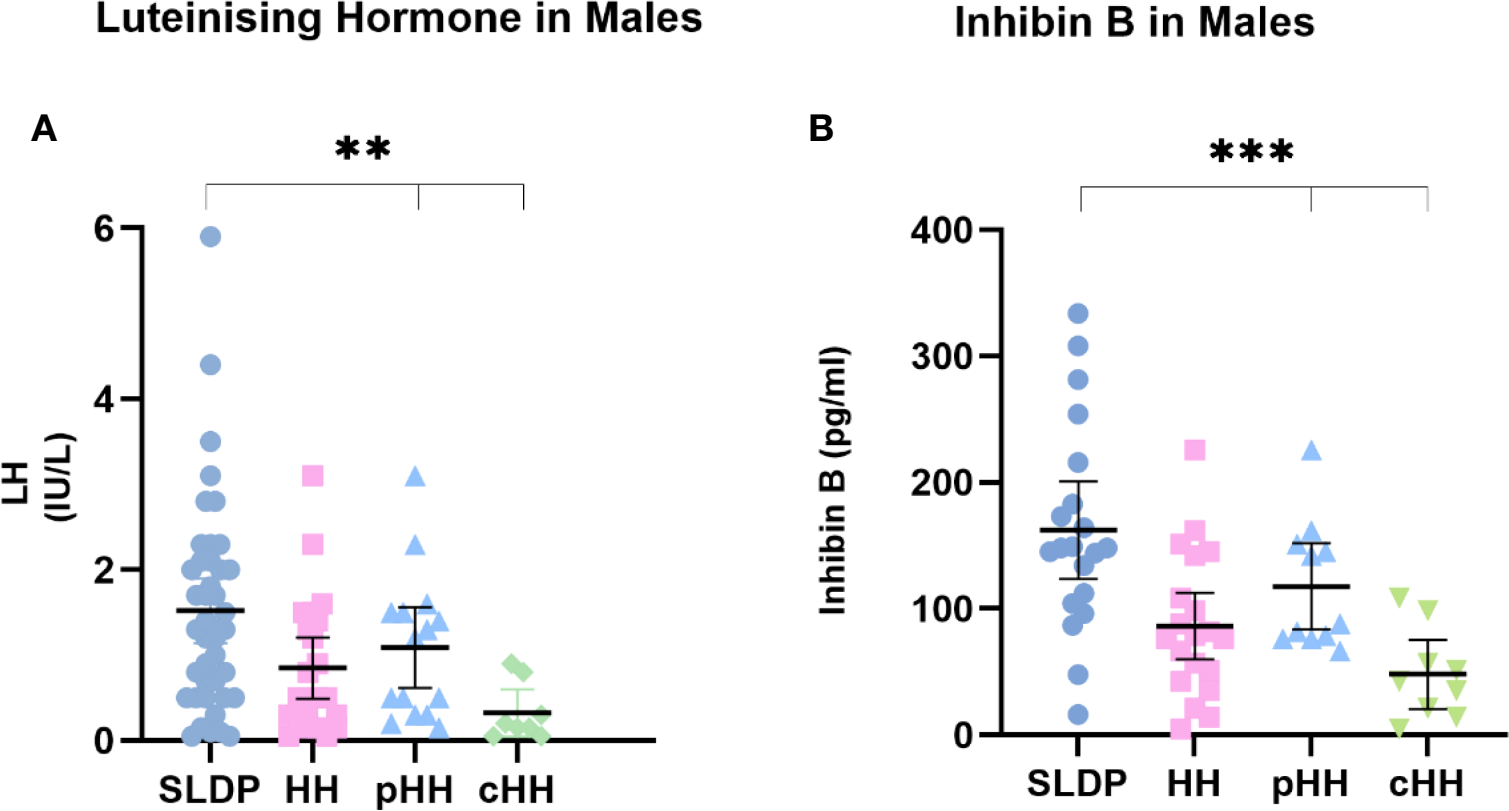
First recorded **(A)** LH and **(B)** inhibin B values for male SLDP (dark blue), HH (pink), pHH (light blue) and cHH (green) groups. Each dot represents an individual patient. Mean and 95% confidence intervals are shown. HH group contains both the pHH and cHH groups. LH, luteinising hormone; HH, hypogonadotrophic hypogonadism; SLDP, self-limited delayed puberty; pHH, partial hypogonadotrophic hypogonadism; cHH, complete hypogonadotrophic hypogonadism. **p value = 0.005, ***p value = 0.001 by one-way Anova.

Whilst mean serum LH in males at presentation was lower in cHH (0.31 ± 0.33 IU/l) than in pHH (1.40 ± 0.73 IU/l) or SLDP (1.56 ± 0.88 IU/l, p=0.01, **Figure 2A**), there was a large degree of overlap (93% SLDP group, 100% of HH group with overlapping LH values, **Figure 3A**). Mean inhibin B was also lower in males with cHH (30.9 ± 36.8 pg/ml) as compared to pHH (117.3 ± 48.8 pg/ml) and SLDP (160.5 ± 54.4 pg/ml, p=0.001) (**Figure 2B**, **Table 4**). However, there was also a large degree of overlap between inhibin B values for the SLDP and HH groups (82% of SLDP and 40% of HH group with overlapping values, **Figure 3B**), suggesting a limited utility also of this parameter, particularly within the range 100-150 pg/ml. There were no significant differences in FSH (p=0.07), testosterone (p=0.36) or AMH concentrations (p=0.81) in male patients at presentation (**Figures 2A, B**), with high degrees of overlapping values (FSH: SLDP 70%, HH 100%; Testosterone: SLDP 58%, HH 100%; AMH: SLDP 100%, HH 86%).

**Table 4:** Biochemical and bone age assessment data at presentation of SLDP and HH patients from the DP cohort.

	SLDP (n=44)		HH (n=25)		P value* (SLDP vs HH)	pHH (n=17)		cHH (n=9)		P value** (SLDP vs pHH vs cHH)
	Mean	SD	Mean	SD		Mean	SD	Mean	SD	
Biochemistry Male, (mean+/-SD)										
Serum LH IU/l (n=45)	1.56	0.88	1.05	0.73	0.054	1.40	0.73	0.31	0.33	**0.005**
Serum FSH IU/l (n=44)	2.46	1.14	1.80	1.12	0.074	2.00	0.75	1.05	1.23	0.07
Serum testosterone nmol/l (n=47)	1.53	1.80	1.54	4.05	0.362	2.52	6.08	0.27	0.16	0.121
Inhibin B pg/ml (n=21)	160.5	54.4	116.6	73.4	**0.015**	117.3	48.8	30.9	36.8	**0.001**
AMH pmol/l (n=14)	195.8	156.7	238.5	231.6	0.805	247.8	252.3	182.7		0.95
Peak LH (n=6)	9.13	4.89	6.27	4.74		5.45	5.44	8.7		
Biochemistry Female, (mean ± SD)										
Serum LH IU/l (n=8)	2.71	2.89	0.56	0.45	0.486	0.35	0.18	1.20		0.462
Serum FSH IU/l (n=8)	5.40	5.97	2.30	0.45	1.000	2.20	0.50	2.60		0.973
Serum oestradiol pmol/l (n=7)	73.8	55.9	33.3	15.0	0.629	30.0	16.5	43.0		0.751
Inhibin B pg/ml (n=2)			19.5	25.7						
AMH pmol/l (n=1)			56.6							

LH, luteinising hormone; FSH, follicle stimulating hormone; AMH, anti-mullerian hormone.

*comparison of SLDP vs HH by student t-test. **comparison of SLDP vs pHH vs cHH by one-way Anova.

Absent p values are due to insufficient numbers for comparison. P values <0.05 are highlighted in bold font.

In female patients, LH (0.56 ± 0.45 vs 2.71 ± 2.89 IU/l) and oestradiol (33.3 ± 15.0 vs 73.8 ± 55.9 pmol/l) concentrations were lower in individuals with HH than those with a final diagnosis of SLDP, but not significantly so (p=0.49, p=0.63 respectively).

### Treatment regimens and time to starting therapy

As expected, a far higher proportion of patients with a final diagnosis of HH received medical therapy for their delayed puberty (**Table 5**). 58% of patients with SLDP, as compared to over 92% of patients with HH, were prescribed hormonal treatment by endocrinology services. All patients with cHH received treatment. Treatment was more commonly received by male than female patients in the SLDP groups (SLDP: male 61.7%, female 40.0%; HH: male 95.5%, female 100%). The mean age at commencing treatment in male patients with SLDP was 15.4 ± 1.2 years, but this was nearly a year later in male patients with HH at 16.2 ± 2.4 years (p=0.21). Additionally, SLDP patients had a documented duration from their initial tertiary services consultation to the beginning of hormonal treatment of 150.4 ± 334 days. In comparison, HH patients took longer to commence therapy (likely related to an increased burden of investigations) with an average of 187.4 ± 390 days (p=0.34).

**Table 5:** Hormonal treatment of SLDP and HH patient groups from the DP cohort.

	SLDP (n=31)		HH (n=24)		P value* (SLDP vs HH)	pHH (n=15)		cHH (n=9)		P value** (SLDP vs pHH vs cHH)
Hormonal treatment (n, %)										
Testosterone	29	93.6	11	45.8		10	66.7	1	11.1	
Oestradiol	2	6.5	4	15.4		2	13.3	1	11.1	
Gonadotrophins	0		2	8.3		0	0	2	22.2	
Testosterone -> Gonadotrophins	0		8	33.3		3	20	5	55.6	
Duration to treatment (days, mean ± SD)										
Male (n=44)	150.4	334.0	187.4	390.2	0.344	60.3	92.4	423.3	604.1	0.112
Female (n=3)			114.3	99.6		171.5	14.8	0		
Age at treatment (decimal years, mean ± SD)										
Male (n=46)	15.4	1.2	16.2	2.4	0.205	16.5	2.2	15.7	2.7	0.402
Female (n=4)	14.6	0	16.9	1.05		16.4	0.7	18.0	0	

*comparison of SLDP vs HH by student t-test. **comparison of SLDP vs pHH vs cHH by one-way Anova. Absent p values are due to insufficient numbers for comparison.

All male patients with SLDP that received medical therapy were treated with testosterone (n=29), mostly started intramuscularly (62.1%, 18/29) rather than orally (20.7%, 6/29) or topically (10.3%, 3/29). Male patients with HH were also mostly treated with testosterone alone (42.3%, n=11), where intramuscular was the commonest mode of delivery (7/11) followed by topical therapy (3/11). One patient was treated only with hCG. The other male patients with HH were treated with a combination of gonadotrophins and testosterone (42.9%, n=9), typically commencing testosterone and then being switched to recombinant (r)FSH and hCG (5/9). Three patients were given rFSH pre-treatment, with hCG then added in (4/9). All female patients (2 SLDP, 3 HH) were treated with 17b-oestradiol either orally or transdermally.

### Progression through puberty differed between patients with SLDP and HH

**Figure 4:**
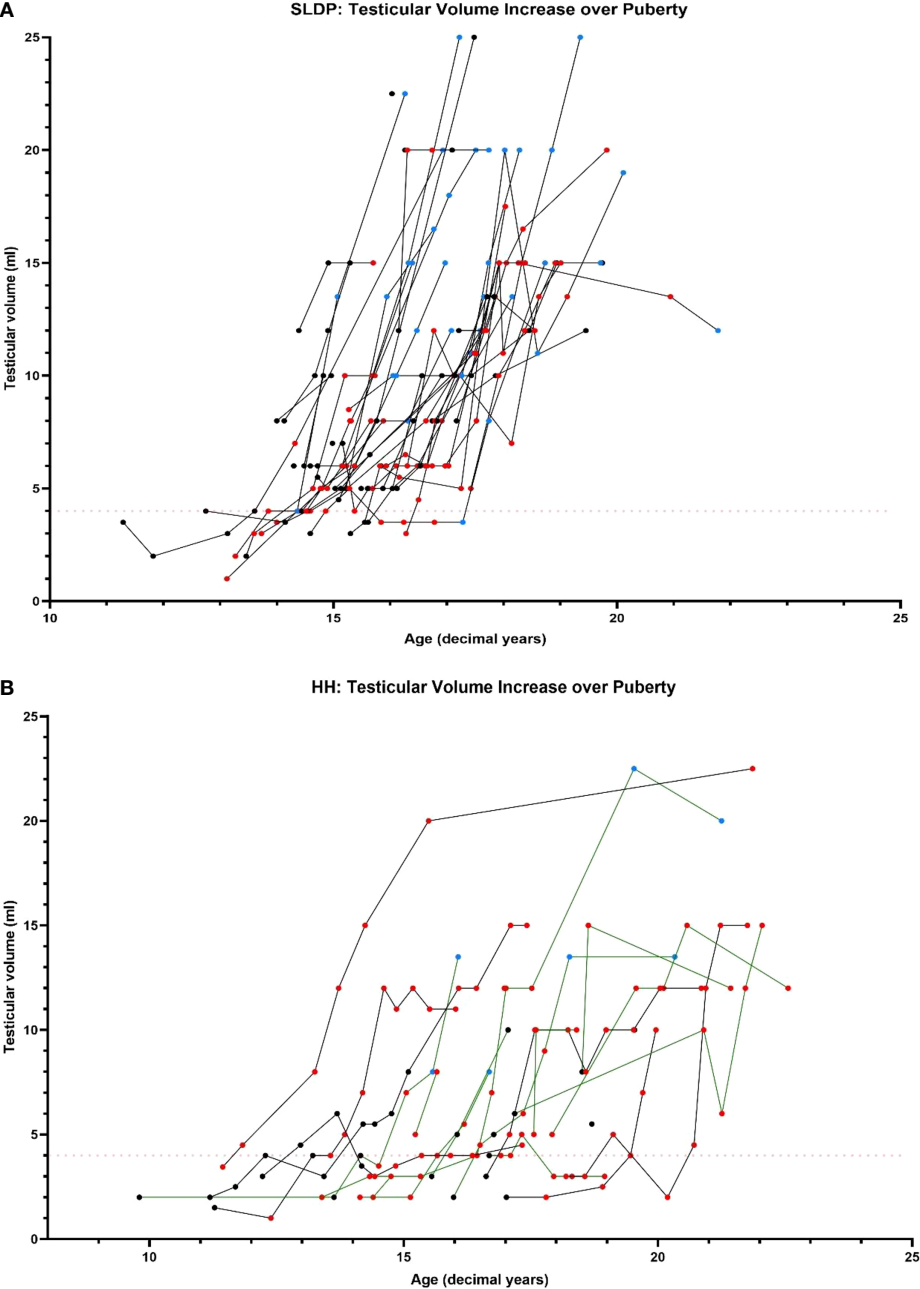
Comparison of testicular volume during adolescence and early adulthood in SLDP **(A)** and HH **(B)** patient groups (partial HH, green lines; complete HH, black lines). Data points shown prior to starting on reproductive hormonal treatment (black), once started on reproductive hormonal treatment (red) and once they had completed treatment (blue). Each line represents one individual. Red dotted line shows testicular volume of 4ml marking onset of puberty.

Male mean age at puberty onset was 14.9 ± 0.87 years in SLDP patients, and 15.6 ± 2.38 years in HH patients (**Figure 4**, p=0.54). There was no difference between age of puberty onset in SLDP patients with spontaneous onset and those receiving hormonal treatment (14.8 ± 0.84 vs 14.9 ± 0.97 years). In comparison, 41.7% patients with HH entered puberty spontaneously at a mean age of 14.9 ± 1.65 years, and 58.3% entered puberty secondary to hormonal treatment at a mean age of 16.0 ± 2.81 years. Age at the end of puberty (achievement of G4) was 16.8 ± 1.06 years in male SLDP patients, and 17.7 ± 2.21 years in male HH patients (p=0.17).

**Figure 5:**
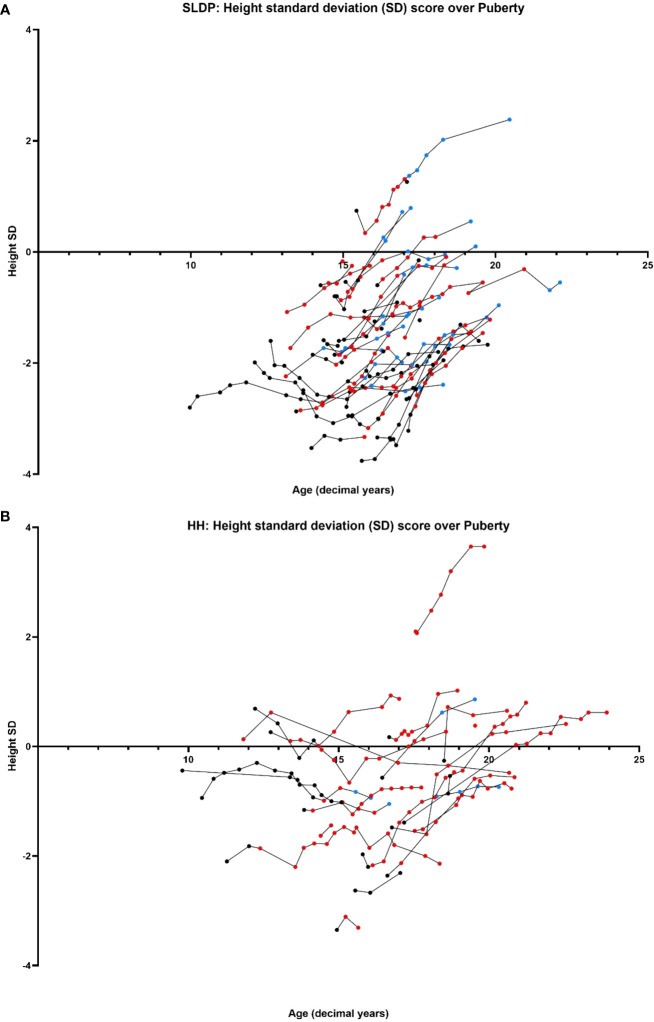
Comparison of age (years) versus height SD score between SLDP **(A)** and HH **(B)** populations. Data points shown prior to starting on reproductive hormonal treatment (black), once started on reproductive hormonal treatment (red) and once they had completed treatment (blue). Each line represents one individual.

Male patients were below mean population height during pubertal progression, and this was particularly striking for the SLDP group (**Figure 5**). At puberty onset, SLDP patients had mean height SD score of -1.73 ± 1.27 and HH patients -0.89 ± 1.09 (p=0.17). At the end of puberty, average height for both groups remained somewhat reduced with male SLDP patients (height SD score -1.46 ± 1.08) shorter than HH patients (height SD score -0.28 ± 1.29, p=0.01, **Figure 5**). SLDP patients, however, showed a higher height velocity at attainment of G4 (4.64 ± 3.23 cm/year) than HH patients (0.91 ± 2.92 cm/year, p=0.004), **Supplemental Figure 1**, with a greater cumulative growth over puberty, **Supplemental Figure 2**. Thus, by the end of growth the difference was not so marked: adult height data were available for 16 male patients in each diagnostic category, with a height SD score (corrected for MPH) of -0.26 ± 0.97 in SLDP patients and -0.16 ± 0.99 in HH patients.

Male SLDP patients were of lower weight (weight SD score -1.11 ± 1.98) than the HH group at puberty onset, particularly when compared to cHH (0.95 ± 1.64) rather than to pHH (-0.51 ± 2.33, p=0.03) patients, and remained lighter at the end of puberty (weight SD score -0.90 ± 2.01) than HH patients (1.17 ± 2.88, p=0.01).

The pattern of testicular development also differed between the two diagnostic groups. Patients with SLDP, both with and without sex steroid therapy, had a relatively uniform increase in testes volume, increasing from 4mls to 15-20ml over a period of 2-3yrs (**Figure 4**). Whether or not patients with SLDP had hormonal treatment was not associated with final testicular volume (p=0.093). In contrast, testes volume increase was more variable in patients with HH and occurred over a longer duration (**Figure 4**, **Supplemental Figure 3**). Greatest recorded testicular volume was significantly higher (p=0.033) in SLDP (14.7 ± 5.52ml, n=46) compared to HH patients (11.8 ± 5.00ml, n=22). There was no significant difference in Tanner P (p=0.807) or A staging at the end of puberty between patients with SLDP and HH (p=0.518).

### LH and inhibin B remained lower in patients with HH, particularly those with cHH, at the end of puberty

At the end of puberty, serum LH remained lower in male patients with HH, most markedly in cHH patients (0.64 ± 0.49 IU/l) compared to pHH patients (2.39 IU/l ±1.58) and SLDP patients (3.04 ± 1.54 IU/l, p=0.008). Serum FSH was not significantly different between the two groups (SLDP, 3.4 ± 1.85 IU/l vs HH, 2.9 ± 1.36 IU/l, p=0.62). Inhibin B also remained lower in males with HH (130.1 ± 85.3 pg/ml) than in the SLDP group (184.6 ± 69.9 pg/ml, p=0.048), despite gonadotropin therapy in 38.7% of the HH patient cohort. Testosterone concentration was not different between the groups at the end of puberty, but this was confounded by the fact that at this point only 17.1% of SLDP patients remained on reproductive hormone treatment as compared to 73.3% of the HH patient group.

Mean age of menarche was 17.8 ± 1.71 years in female patients with HH and 15.6 ± 0.82 in females with SLDP.

## Discussion

Delayed pubertal onset or development is a common presentation to adolescent endocrine services, and the differentiation of SLDP from the more severe condition of congenital HH is often challenging. Whilst the expected frequency of SLDP is far higher than that of HH in children presenting with delayed onset of puberty aged 14-15 years, by the time an individual is 17-18 years old the likelihood of a diagnosis of HH is greatly increased (7). With long-term follow up the diagnosis can be made clinically, but in adolescence the features of pubertal delay and delayed skeletal maturation, in conjunction with low or undetectable sex steroid and gonadotropin concentrations, are present in both conditions.

The diagnostic complexity is particularly true for patients with incomplete or partial GnRH deficiency, who may have entered puberty and then stalled in their pubertal progression, and whose clinical and biochemical parameters may be intermediate between those of SLDP and complete HH (3, 4, 7). Indeed, it is exactly this group of patients with severe delayed puberty but without clear ‘red flag’ signs for the diagnosis of congenital HH that present that greatest clinical challenge.

The lack of a gold standard test to differentiate SLDP from HH can lead to delay and frustration for patients and clinicians alike, with the need for extensive and costly investigations which can take months or even years. Recent exciting research into the use of kisspeptin to differentiate these two pathologies have given hope for improved biochemical diagnosis in the future, but these tests are not currently available outside of these clinical trials (22, 23). Moreover, accurate diagnosis is important as this is instrumental in directing and optimising care and improving the overall quality of life of the affected individuals (24). Those patients diagnosed with congenital HH during adolescence can have the option to induce puberty with gonadotropins or in certain centres GnRH, which can allow testicular development and optimise the potential for future fertility (25). In parallel, a clear diagnosis of SLDP can provide reassurance for this patient group that puberty will ensue and that, if required, a course of sex steroids will be sufficient to induce pubertal onset, leading to a reduction in psychological stress and avoidance of prolonged investigations (26).

In view of this, our present study sought to carefully review the clinical, auxological, biochemical and associated features of a UK cohort of patients with delayed puberty, consisting entirely of patients who had been followed up to final diagnosis of either SLDP or congenital HH. Our aim was to analyse the key differences between these two disease groups both at presentation, when potential for diagnosis may be limited, and during progression through puberty, when further clarity as to the underlying aetiology of the pubertal delay may become more evident.

In keeping with the literature, in this cohort SLDP was the predominant form of DP identified, with two-thirds of our patients having this as their final diagnosis. Indeed, the proportion of patients with HH in our cohort was higher than in some previous studies (7), likely reflecting the fact that our study recruitment sites are mainly tertiary or quaternary paediatric endocrine centres, with more referrals of extreme and complex cases. Our population was also predominantly male in both diagnostic groups, as is well documented to be the norm for adolescent DP cohorts (4), and thus provided more insights into male puberty in these conditions. SLDP is well-recognised to be a commonly inherited condition with strong genetic drivers (27), and whilst a family history of delayed puberty was seen more frequently in patients with SLDP, a family history of delay was also seen in patients with HH, particularly with pHH. It has been documented previously that patients with congenital HH may have family members with SLDP (28), and this suggests a note of caution in the potential for false reassurance in a patient with a positive family history of DP. Moreover, increasing numbers of patients with congenital HH, particularly pHH, are receiving successful fertility treatment and having children, and thus a careful history taking of the nature of pubertal delay and treatment in parents is needed in these families.

On their first presentation to endocrinology services, both SLDP and HH patients were of below average height and weight for age, with SLDP significantly further below the mean for population in both categories. Mid-parental height for patients with SLDP was not significantly different from the HH group; thus, the SLDP patients did not present with low height SD just because their parents are short. Bone age was delayed in both groups, more so in the SLDP group, suggesting that the low presenting height SD score of the SLDP patients is likely related to the constitutional element of the pathophysiology of this condition, with slower growth over a number of years preceding adolescence. This echoes previous findings of delayed growth patterns in SLDP compared to HH in the first 2 years of life, far prior to pubertal onset (16). Interestingly, the patients with a final diagnosis of pHH were similar to those with cHH with respect to height at presentation or bone age delay, suggesting this may be a parameter which may aid differentiation of pHH from SLDP.

Males with HH, and particularly cHH patients, had a more frequent history of particular red flags - bilateral cryptorchidism and micropenis – which indicate gonadotrophin deficiency during late gestation and the mini-puberty period of infancy. In contrast, mini-puberty is thought to occur normally in patients with SLDP. Postnatal activation of the HPG axis during mini-puberty in male infants is responsible for testes and penile growth after birth, with Sertoli cell proliferation, as well as promoting testicular descent (29). Whilst mini-puberty is also likely to be important in females for ovarian development, its effects are not so evident clinically as they are in males (30). The low inhibin B concentrations seen in patients with HH also reflects this lack of pre-pubertal HPG axis stimulation secondary to GnRH deficiency, with cHH patients having markedly lower inhibin B concentrations in adolescence (31). Inhibin B is part of the transforming growth factor-β family and is produced by ovarian granulosa cells in females and by testicular Sertoli cells in males. Lack of mini-puberty in patients with severe GnRH deficiency thus results in the classical cHH phenotype with low inhibin B, with small testicular volumes in males and associated features of micropenis and cryptorchidism. Whilst the difference in frequency of micropenis between patients with SLDP and HH reached significance, the same was not true for cryptorchidism, likely reflecting the low numbers of patients seen with this feature (3 patients in each group).

In contrast, those patients with pHH who have had some degree of postnatal HPG axis activity may have none of these features and may be much more difficult to distinguish from patients with SLDP. Indeed, in our cohort, patients with pHH had an intermediate phenotype between SLDP and cHH for frequency of micropenis, testes volume and inhibin B at presentation. Whilst for patients with cHH, 75% of patients had LH values and 79% had inhibin B values below previously suggested cut-offs for diagnosis of congenital HH, of 0.5mIU/L (32) and 61pg/ml (33) respectively, inhibin B in all individuals in the pHH group was higher than this threshold value. Thus, inhibin B in our cohort was not a useful test for the diagnosis of HH in males with the partial form of the disease. From our study, the clearest distinguishers of pHH from SLDP at presentation were height SD score (lower in SLDP) and the presence of anosmia and micropenis (less frequent in SLDP).

No associations were seen in other biochemical markers proposed for the differentiation of SLDP from congenital HH, including FSH and AMH (18, 34), although AMH was only available in small numbers of patients in our cohort.

Beyond initial presentation, patients with SLDP remained significantly growth delayed at pubertal onset, whereas those with HH (particularly cHH) had caught up, perhaps due to differences in timing of hormonal treatment initiation. This height deficit in patients with SLDP remained evident throughout puberty, although notably SLDP patients had a higher height velocity at attainment of G4 suggesting that they may not yet have reached their adult height. Previous studies have reported conflicting evidence as to whether SLDP patients eventually achieve their full genetic height potential (12), with some suggesting that they achieve an average BMI (16) and others indicating that they fail to achieve their target height (35).

Patients with HH entered puberty later than those with SLDP and achieved (treatment-induced) end of puberty status nearly a year later. Men with HH had reduced final testes volume and serum inhibin B, despite gonadotropin therapy in more than a third of this diagnostic group. It is very likely that with greater availability of gonadotropin therapy for male patients with HH, particularly the use of pre-treatment with rFSH prior to hCG therapy, testicular development can be further optimised in this treatment group (36).

Limitations of this study include a wide variation in age and Tanner stage at participant presentation; although in this way the study reflects the real-world clinical setting. Our data included a limited sample size for particular parameters including AMH, where data on this were missing for a large number of cohort participants, as well as inter-observer variability across institutions (for example regarding Tanner staging), and limited sample sizes of female patients. Differing treatment regimes were used across the cohort, with some patients with HH receiving sex steroids and others gonadotropin therapy to achieve medication-induced puberty. Some data were also lost at the point of completion of puberty, in part due to long intervals between clinic appointments and patients discharged or lost to follow-up.

In conclusion, although no definitive markers yet exist to conclusively diagnose these two conditions, this study has highlighted several phenotypic markers that may be helpful to distinguish HH from SLDP at presentation; namely lower height and weight, and higher testicular volume, serum LH and inhibin B in those with SLDP. In the case of height, weight, LH and inhibin B, these relationships persisted through puberty. To this end, it would be useful to establish these parameters as important baseline investigations across different institutions. However, in view of the high degree of overlap for many of these biochemical tests, their discriminatory power as individual biomarkers is limited, particularly for partial forms of HH. Further research is therefore warranted into optimisation of measurement intervals and cut-off values within the development of a framework or scoring system incorporating clinical, biochemical and genetic features to aid and streamline clinical decision-making in diagnostically complex cases, in order to improve diagnostic accuracy and earlier treatment optimisation.

## Data availability statement

The original contributions presented in the study are included in the article/**Supplementary Material**. Further inquiries can be directed to the corresponding author.

## Ethics statement

The studies involving humans were approved by the London–Chelsea NRES committee and the UK NHS Health Research Authority (13/LO/0257). The studies were conducted in accordance with the local legislation and institutional requirements. Written informed consent for participation in this study was provided by the participants’ legal guardians/next of kin.

## Author contributions

SH conceptualised and designed the study. YA, VK, KY, KB, GB, MD, PD, CH, MM, MK, GP, SS, PS, HS, RW and SH collected the clinical data. YA, VK and SH analysed the data. YA, VK and SH wrote the original draft. LD, MD, MK critically reviewed the manuscript. YA and SH edited the final version. All authors had final responsibility for the decision to submit for publication.
