# Supplementary material for: Key features of puberty onset and progression can help distinguish self-limited delayed puberty from congenital hypogonadotrophic hypogonadism
Source: Front Endocrinol (Lausanne). 2023 Aug 28;14:1226839. doi: 10.3389/fendo.2023.1226839 (PMC10493306; doi:10.3389/fendo.2023.1226839)
Supplement: Supplementary file 1 [file DataSheet_1.docx]

**Supplemental Appendix**

*
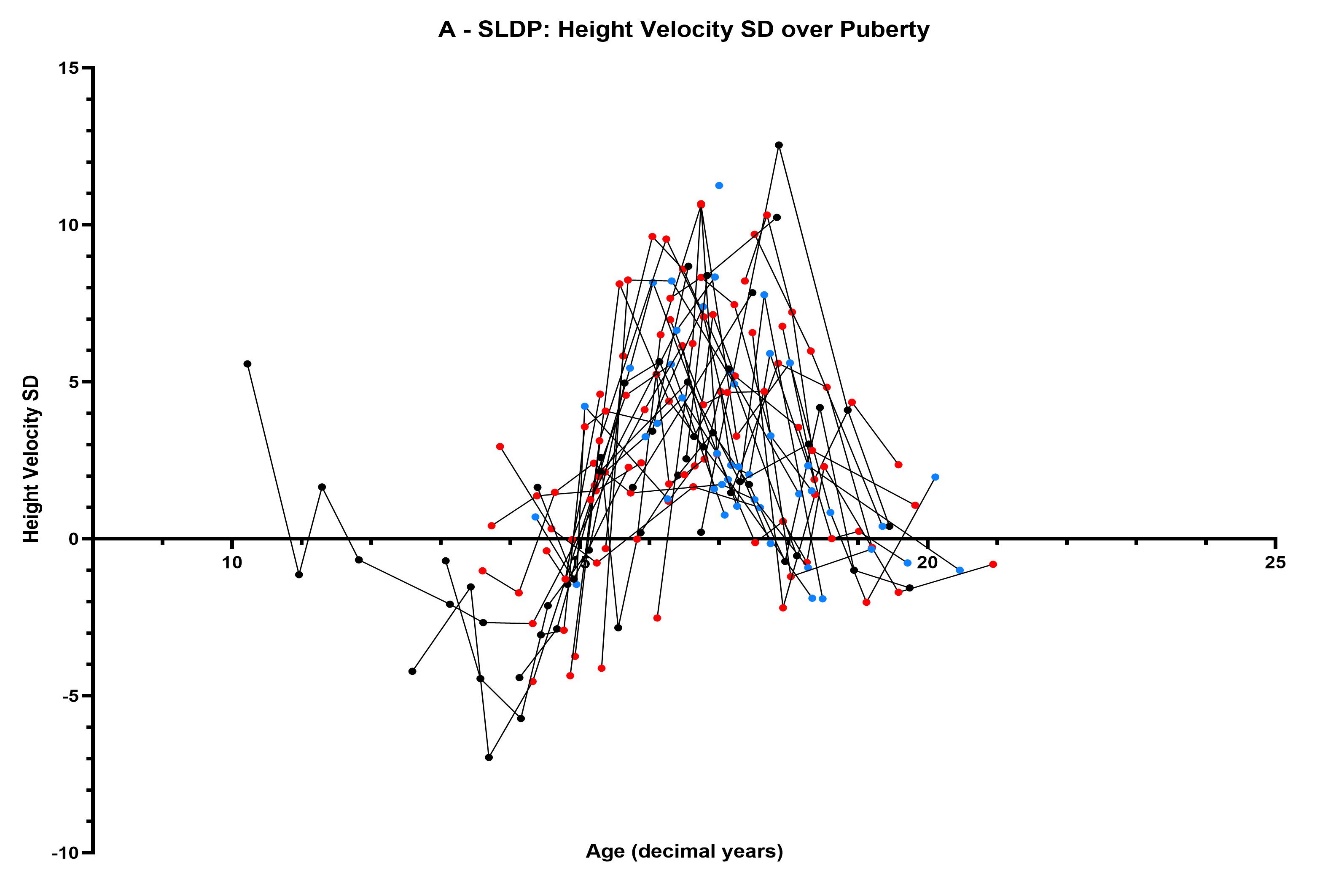
*

*
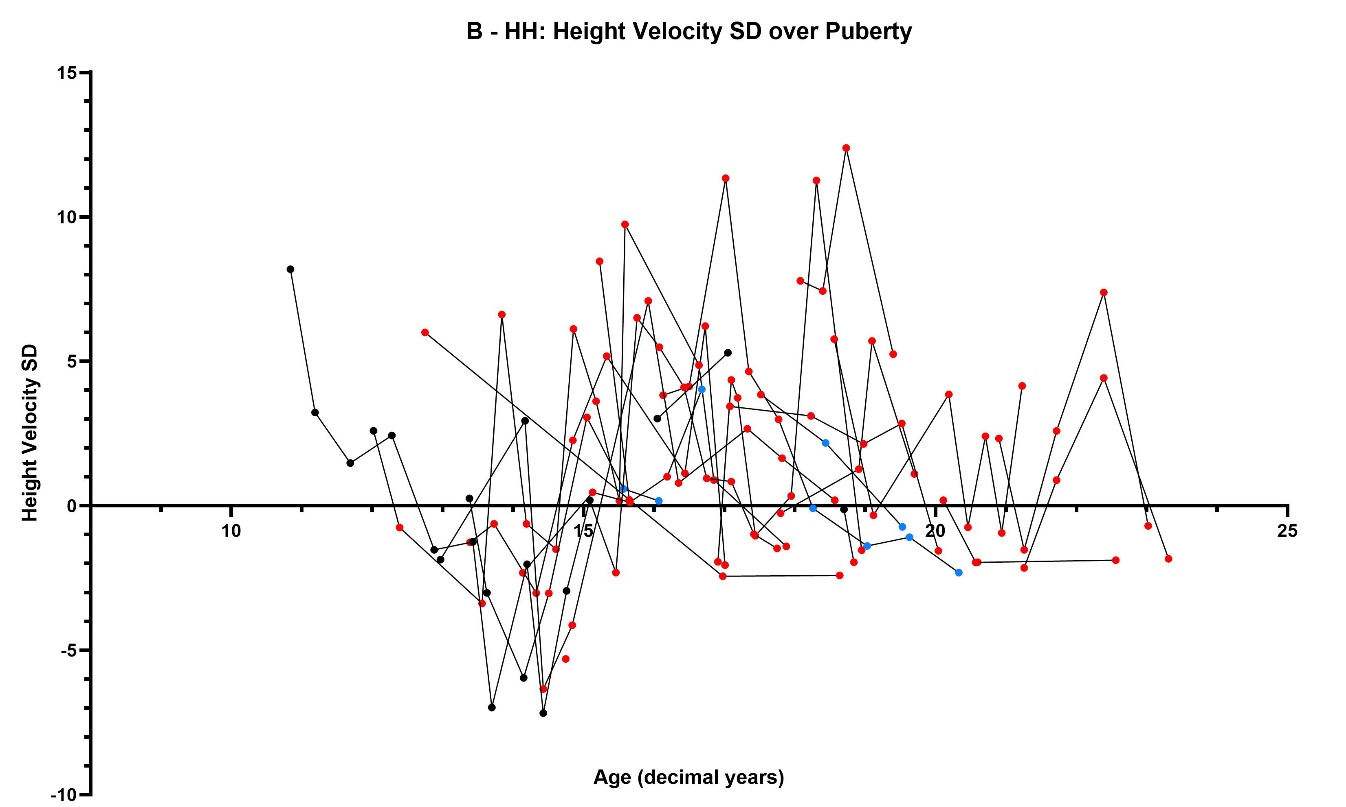
*

***Supplementary Figure 1.*** *Comparison of height velocity SD at varying age in SLDP (top panel) and HH (bottom panel) diagnostic groups. Data points shown prior to starting on reproductive hormonal treatment (black), once started on reproductive hormonal treatment (red) and once they had completed treatment (blue). Each line represents one individual.*

***
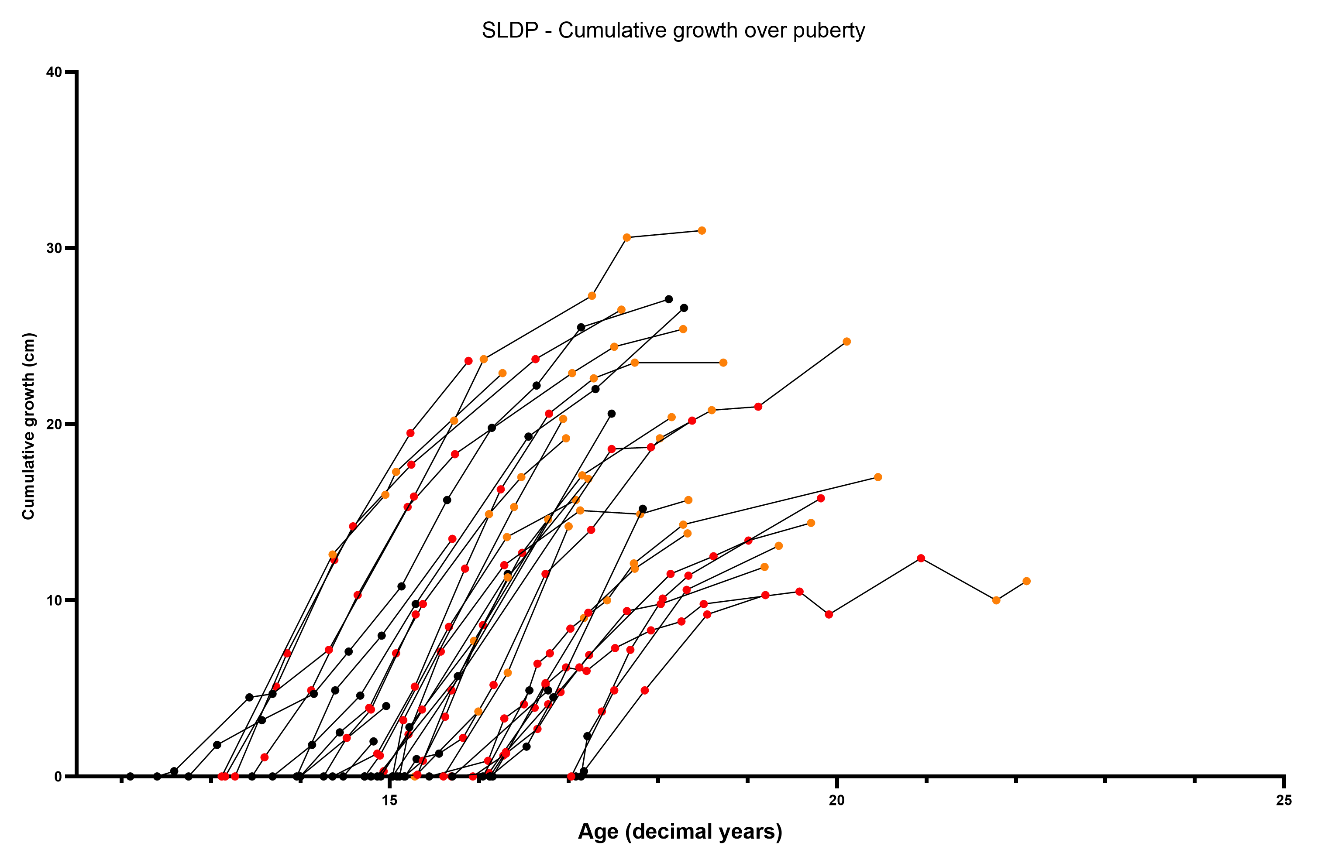

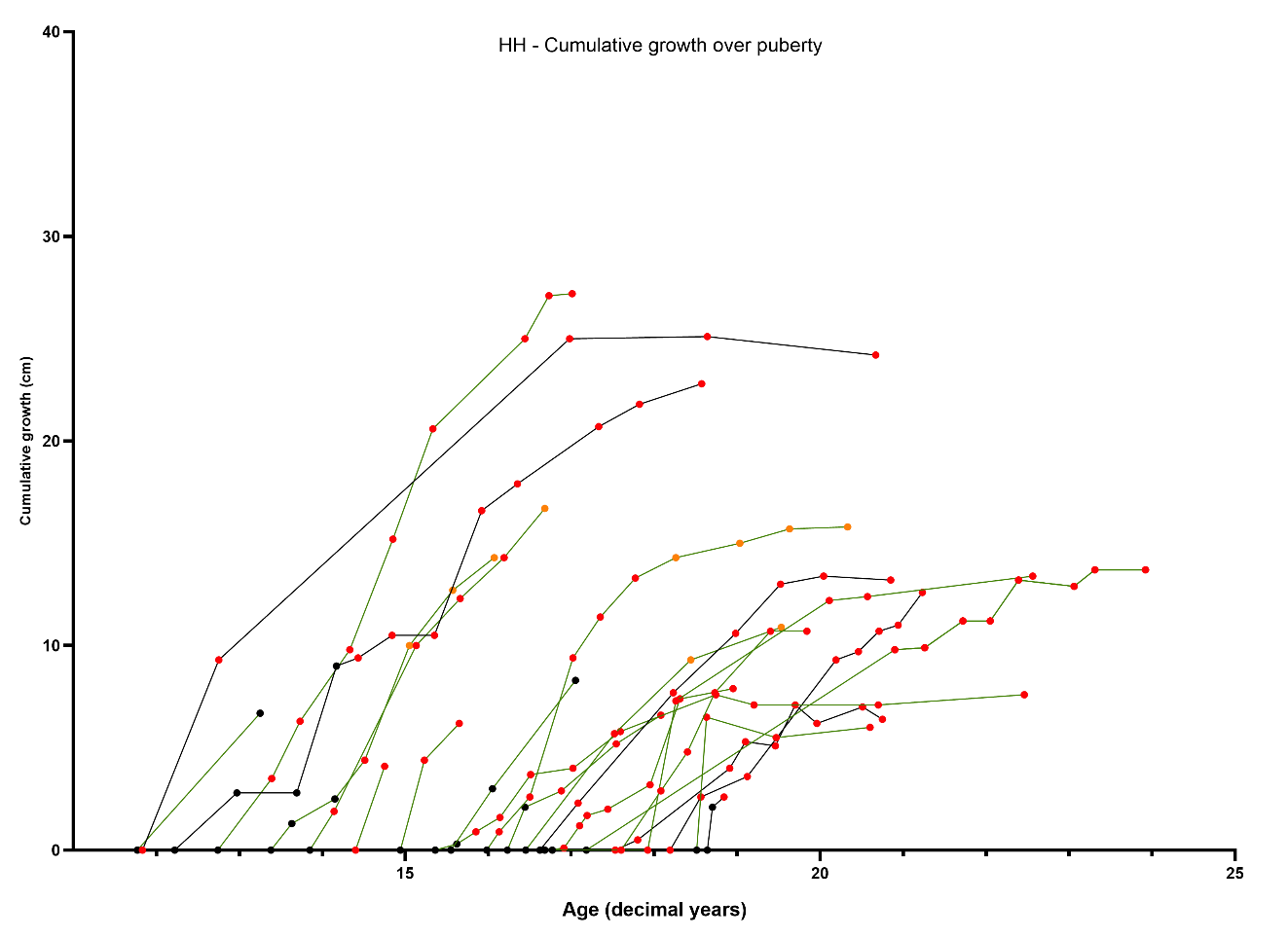
***

***Supplementary Figure 2.***  *Comparison of cumulative growth in cm over puberty in male SLDP (top panel) and HH (bottom panel) diagnostic groups. Data points shown prior to starting on reproductive hormonal treatment (black), once started on reproductive hormonal treatment (red) and once they had completed treatment (blue). Each line represents one individual.*


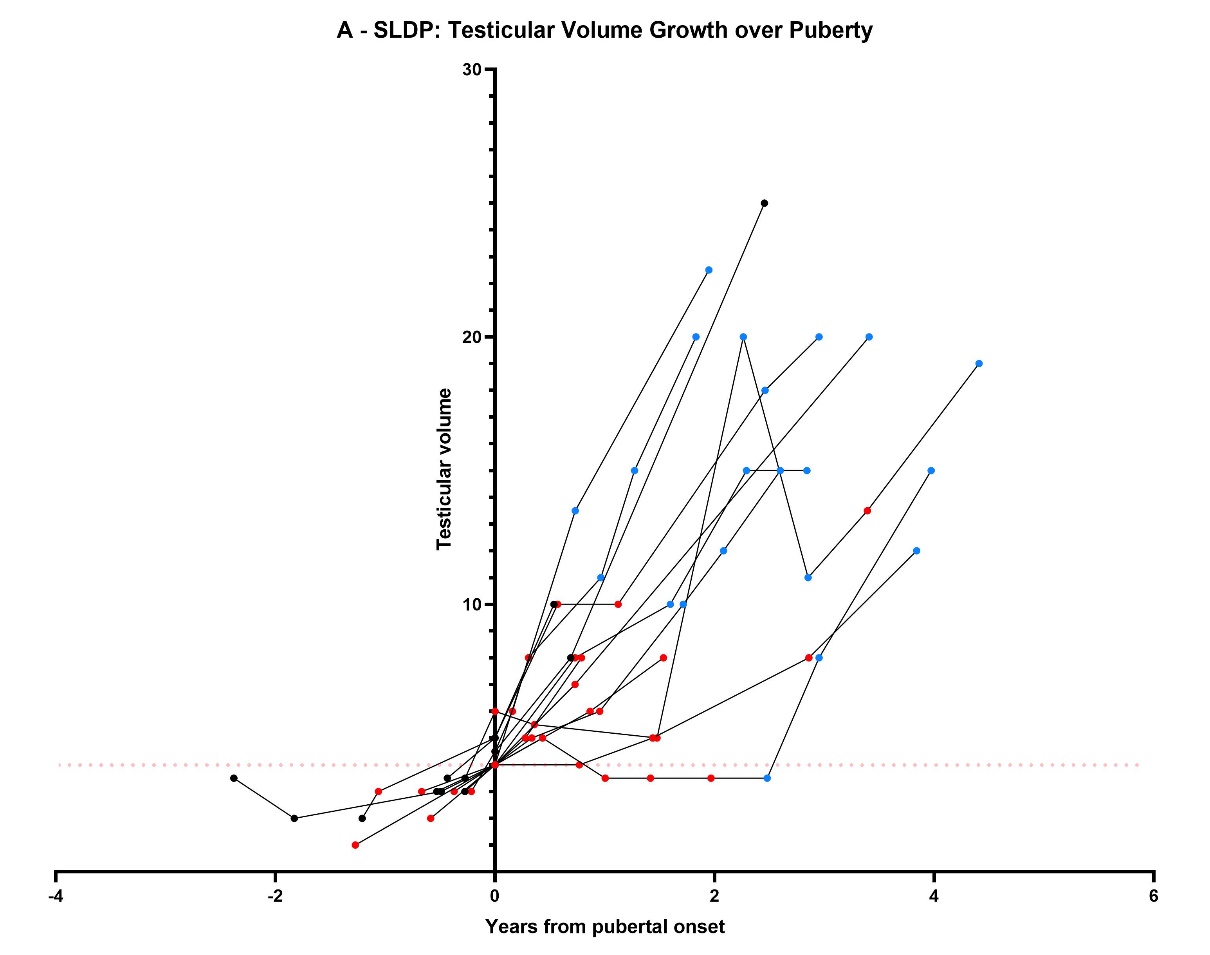


*
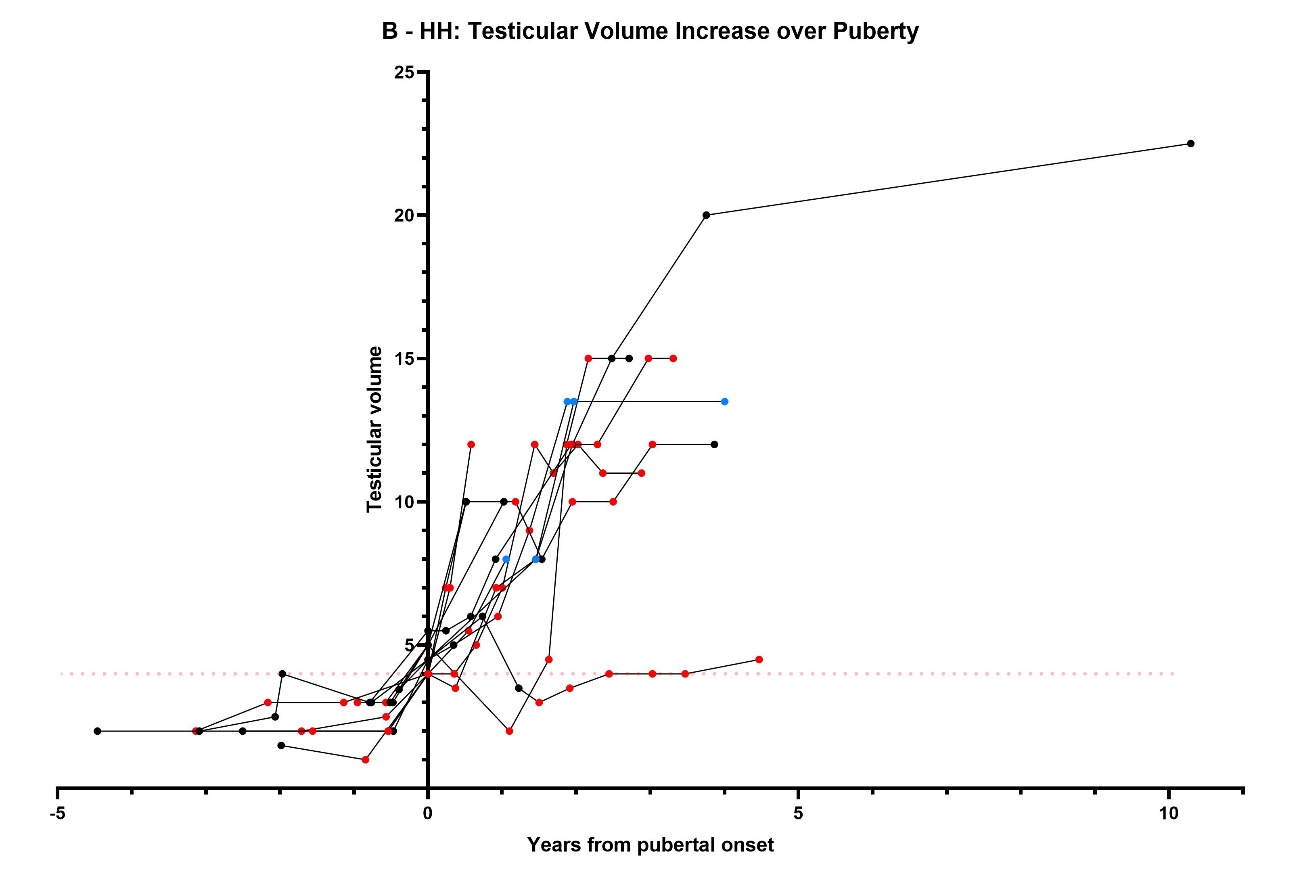
*

***Supplementary Figure 3.*** *Comparison of years from puberty onset and testicular volume in male SLDP (top panel) and HH (bottom panel) diagnostic groups. Data points shown prior to starting on reproductive hormonal treatment (black), once started on reproductive hormonal treatment (red) and once they had completed treatment (blue). Each line represents one individual.*
